# A Majority of Admitted Patients With Ruptured Abdominal Aortic Aneurysm Undergo and Survive Corrective Treatment: A Population-Based Retrospective Cohort Study

**DOI:** 10.1007/s00268-016-3705-9

**Published:** 2016-08-22

**Authors:** R. Hultgren, Sayid Zommorodi, Moa Gambe, Joy Roy

**Affiliations:** 1 Department of Molecular Medicine and Surgery, Karolinska Institutet, Stockholm, Sweden; 2 Department of Vascular Surgery A2:01, Karolinska University Hospital, Stockholm, Sweden; 3 Section for Vascular Surgery, Department of Surgery, Södersjukhuset, Stockholm, Sweden

## Abstract

**Background:**

Abdominal aortic aneurysm (AAA) is an asymptomatic, potentially lethal condition predominantly found in elderly. The mortality is 100 % if rupture occurs and left untreated, but even in treated patients the mortality is substantial. Female sex and treatment with open repair rather than endovascular aortic repair (EVAR) have been reported to negatively affect outcome. The objective was to describe the contemporary care and outcome of all treated and untreated patients with ruptured AAA (rAAA) admitted to hospital.

**Method:**

Population-based retrospective investigation, including all patients admitted to the emergency departments within Stockholm County diagnosed with rAAA 2009–2013. All identified patients’ charts (*n* = 297) were analyzed; the study cohort includes 283 verified patients.

**Results:**

Men were in majority [214 (76 %), 69 (24 %) women] and were younger than women (78 vs 82 years, *p* < 0.001). A majority of patients were treated (212/283, 75 %), a similar proportion of women and men. Untreated patients had a higher mean age (84 vs 77 years, *p* < 0.001). The proportion treated with EVAR was 27 %, and they were older than OR treated (79 vs 76 years, *p* = 0.043). Forty-seven percentage of patients admitted with rAAA survived 30 days, and 62 % of treated patients survived 30 days. The 30-day mortality for women and men was similar.

**Conclusions:**

Our results and other contemporary series show a shift toward a higher rate of treated patients with rAAA, and improving outcomes, similar for women and men. The increased use of EVAR contributes to this improvement in short-term outcome. High age influences the willingness to treat patients with rAAA.

## Introduction

**Fig. 1:**
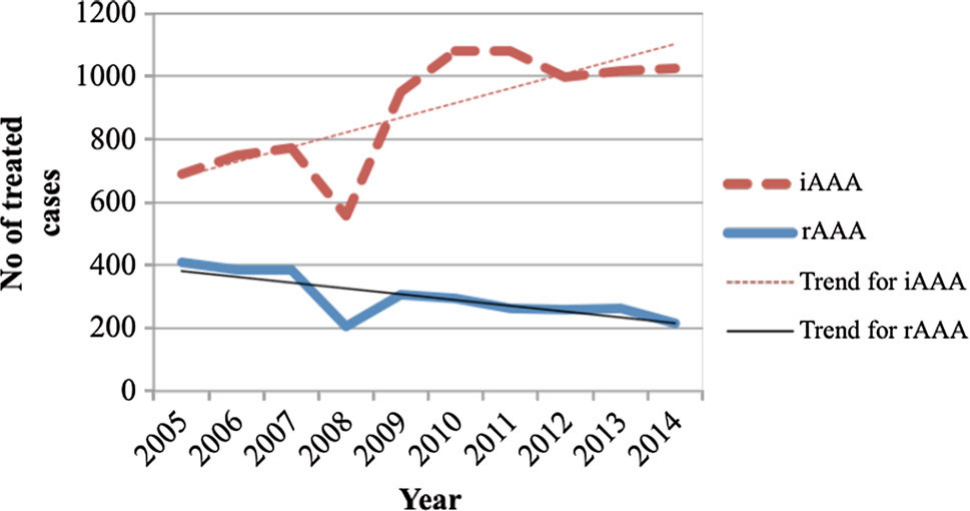
Annual number of elective and ruptured patients treated in Sweden 2005–2013, from SWEDVASC, changes in the Web-based program was performed in 2008, which influences the number of all registered cases

Abdominal aortic aneurysm (AAA) is an asymptomatic, not uncommon and potentially lethal condition predominantly found in persons above 50 years of age. Patients with ruptured AAA, left untreated, have a 100 % fatality rate. The mortality is substantial even in treated patients with ruptured AAA (rAAA), ranging from 20 to 60 % [1–3]. Interestingly, both AAA prevalence and the number of patients treated for rAAA have declined the past 20 years; such a trend is reported in the Swedish national registry parallel to an increment of elective repair (Fig. 1) [1, 3, 4]. A further decline is expected due to the population-based screening program for AAA in elderly men introduced in our region [1, 3, 5–8].

Most studies have focused only on the treated patients, and historically only a few studies have addressed and included the persons admitted but left untreated, which indeed does affect the view on patient care of this group [2, 9–11]. Treated women, both in series of ruptured and elective cases, have been reported to have a worse outcome than men. This can correlate with their higher age when treated, poorer morphology or different distribution of risk factors [1, 12–16] A risk of withholding corrective treatment in women with rupture compared to men has been described: 73 % of men treated versus 56 % of women, also confirmed in a single-center study from Australia (fewer women were treated 37 vs 63 %) [17]. The strongest predictor for death due to most surgical procedures, as well as rAAA, is old age [1, 12, 18–22]. It is not certain how influential the patient’s age is on the decision to offer corrective treatment to rAAA patients. Mortality is higher after OR than EVAR according to retrospective studies [12, 21, 23, 24], and this is also reported in the Swedish Vascular Registry [3]. EVAR is a less invasive method than OR, with a lower perioperative complication rate, but randomized trials have failed to show a difference in short-term mortality [25–27].

The objective with this population-based investigation was to report differences in the contemporary care of all treated and untreated patients with rAAA admitted to a hospital with special consideration of age and gender.

## Materials and methods

### Study population and settings

There are two centralized vascular departments serving the county with emergency and elective vascular care: Karolinska University Hospital and Stockholm South General Hospital (Södersjukhuset), which together cover 20 % of the Swedish population, including care of patients on the Island of Gotland. Patients operated outside of Stockholm County were excluded from this analysis.

All patients admitted to the emergency departments of the seven county hospitals within Stockholm County and Island of Gotland, and diagnosed with rAAA according to revision 10 of the International Classification of Diseases (ICD 10: I71.3) from January 2009 to December 2013 were identified. All hospitals have electronic daily updated chart systems, and the healthcare system is based on a delivery of the patients’ care (date for hospital stay, diagnosis, operations and the unique personal number of each inhabitant) which will then allow reimbursement to the hospital accordingly. This financial hospital system minimizes events of care that are unregistered. Surgery, medicine, cardiology and emergency departments were included in the requisition, to minimize missing rAAA patients admitted at other departments.

### Data collection

All identified patients admitted to the hospitals were identified by their unique personal identity numbers at each hospital (*n* = 297). Twelve patients were excluded; because of previous aortic surgery (*n* = 10) and mycotic rAAA (*n* = 2). Two patients could not be identified due to invalid personal registration numbers.

### Definitions

AAA was defined as an aneurysm with an infra-renal proximal limit. rAAA was verified as the diagnosis mentioned explicitly in medical records, with typical rupture verified by radiology or at intervention, or in an autopsy report. Hospital stay and ICU stay were calculated from date of admittance and date of discharge. AAA diameter was defined as the largest diameter of the ruptured aneurysm on CT or ultrasound. Type of surgery was defined as either OR or EVAR. The group definition “Cardiac disease” includes: former or current heart failure, angina pectoris, myocardial infarction or arrhythmia mentioned in medical records. Smoking was defined as past or current smoking, from medical records. Preoperative mortality was defined as death before start of operation. Perioperative mortality was defined as death during operation, including anesthesia. 30-day mortality included perioperative and postoperative death within 30 days after surgery. There were some missing values, such as smoking habits, which contribute to lower numbers in the presented tables. Height and weight were used to calculate body mass index (BMI).

### Statistics

Continuous variables, with a normal distribution (age, AAA diameter and anthropometrical variables), are presented as mean ± standard deviation (SD). Normal distribution of continuous variables was tested according to Shapiro–Wilk’s, where *p* > 0.05 indicates normality; independent *t* test was used for continuous variables. Categorical values were presented as absolute numbers and percentages. Pearson’s Chi-square test was used for categorical variables. SPSS^®^ version 22 (IBM^®^, Armonk, New York, US) was used for statistical analysis and calculation. Level of significance was set at *p* ≤ 0.05.

#### Ethical permission and reporting

The study was approved by the Regional Ethical Review Board in Stockholm. Registration number: 2013/1277-31/3. The reporting of this study conforms to the Strengthening the Reporting of Observational Studies in Epidemiology (STROBE) statement [28].

## Results

### Patient characteristics

**Fig. 2:**
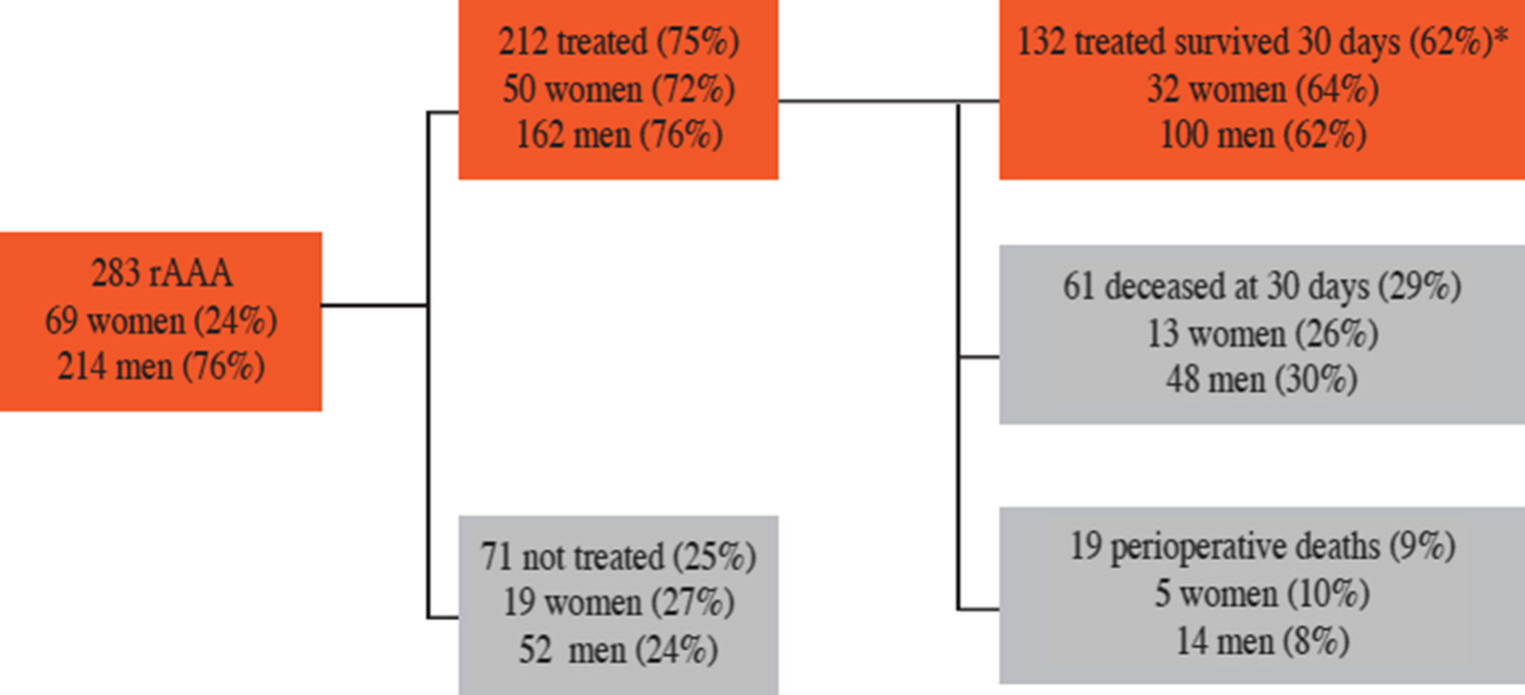
All admitted patients with rAAA, and mortality within 30 days for all women and men during 2009–2013. *Red boxes* show surviving patients, *gray boxes* show the deceased. *percent of treated cohort, *n* = 212

The study cohort is based on 283 patients diagnosed with rAAA, 214 (76 %) men and 69 (24 %) women (Fig. 2; Table 1). Women were older (82 vs 78 years, *p* < 0.001) compared to men (Table 1). The distribution between the age groups was different for women and men, and a majority of women were older than 80 as compared to men (69 vs 48 %, *p* = 0.005). The youngest woman was 67 years, the youngest man was 48, and 20 men were admitted with rAAA below 65 years of age (9 % of all men). Men had higher body mass index and creatinine clearance as compared to women; most other comorbid conditions were similar. The ICU stay for patients admitted after treatment was similar for women and men, and the mean stay was 4 days (Table 1).

**Table 1:** Characteristics, treatment and outcome variables for all included women and men

Variable	Men *n* = 214	Women *n* = 69	*p*
Age (years)	77.56 ± 9.26	82.14 ± 6.57	<0.001
BMI^b^	28.4 (16.8)	22.4 (2.9)	0.002
AAA diameter (mm)^d^	82.7 ± 20.23	73.6 ± 15.98	0.006
Previously known AAA	58 (27.1)	27 (39.1)	0.058
Hypertension	126 (58.9)	49 (71.6)	0.19
Diabetes mellitus	27 (12.6)	8 (11.6)	0.961
COPD	27(.12.6)	15 (21.7)	0.18
Cardiac disease	81 (37.9)	21 (30.4)	0.475
Ever smoker^a^	82/110 (74.5)	24/34 (70.6)	0.752
Pre-op creatinine^c^	121.4 (51.4)	107.8 (42.4)	0.089
Operation rate	162 (75.7)	50 (72.4)	0.625
ICU stay all (days)	4.38 (7.77)	3.27 (6.05)	0.287
30-Day mortality all admitted	114 (53)	37 (54)	0.756
Treated	162 men	50 women	
Mean age	75.7 (8.68)	80.7 (6.15)	<0.001
EVAR rate (% of operated)	41 (25.3)	17 (34.0)	0.511
Hospital stay (days) for treated	13.7 (18.2)	11.3 (10.5)	0.245
ICU stay (days)	5.80 (8.52)	4.57 (6.05)	0.278
Perioperative mortality	14 (8.9)	5 (10.4)	0.815
30-Day mortality all treated	62 (38)	18 (36)	0.860

Presented as numbers (%) and mean (standard deviation)

^a^Missing cases: 35 women and 104 men, classified as unknown smoking history, ^b^kg/m 2, ^c^micromol/l

^d^According to primary assessment by radiologist (134 missing assessments)

Mortality rates (preoperative, perioperative, postoperative) were similar in men and women, even though women were 4.5 years older (Table 1). Overall, 47 % of patients admitted to hospital with rAAA will survive 30 days, and 62 % of patients subjected to treatment will survive 30 days (Table 1; Fig. 2).

### Treatment and outcome

#### Treated

The intervention rates were similar in women and men (72 vs 76 %, *p* = 0.625; Table 1). The treated women had a higher mean age than treated men (Table 1). Among the treated patients, men had a trend toward a higher prevalence of cardiac disease than women [61/162 (38 %) vs 11/50 (22 %) in women, *p* = 0.07]. Diabetes, hypertension and COPD were similarly distributed (data not shown).

**Fig. 3:**
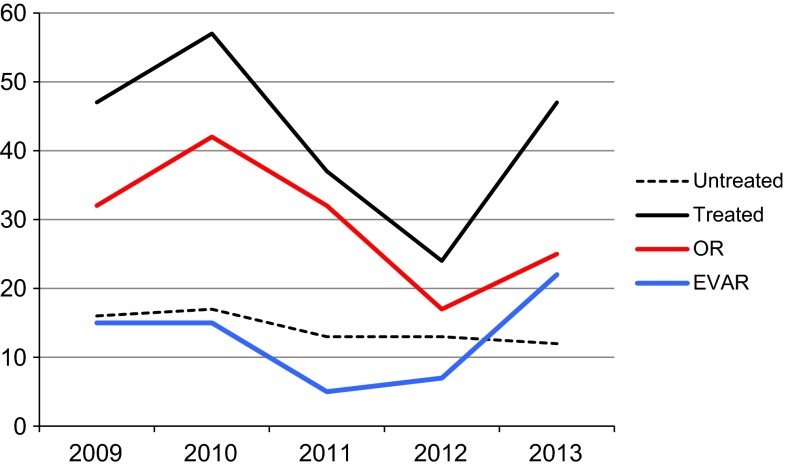
Annual number of untreated and treated patients, and the number of treated patients with EVAR or OR 2009–2013

The proportion of patients treated with EVAR was 27 %, similar for women and men (34 vs 25 %, *p* = 0.51; Table 1). The use of EVAR increased during the later observed time period (Fig. 3). The EVAR-treated patients were older than OR-treated patients (79 vs 76 years, *p* = 0.043; Table 2). The comorbidity profile was similar between the groups (Table 2). The lowest preoperative systolic BP was found in the OR group.

**Table 2:** Treated with EVAR versus OR

Variable	OR *N* = 154	EVAR *N* = 58	*p*
Mean age (SD)	76.2 (7.96)	78.8 (9.34)	0.043
Previously detected	36 (23)	14 (24)	0.907
Women	33 (21)	17 (29)	0.223
Hypertension	99 (64)	34 (59)	0.45
Heart disease	52 (34)	20 (34)	0.63
COPD	18 (12)	13 (22)	0.117
Creatinine	115.4 (44.0)	126.5 (63.3)	0.26
Blood pressure, lowest before^a^	74.12 (41.1)	96.30 (39.1)	<0.001
ICU stay (days)	6.3 (8.81)	3.49 (4.93)	0.006
Hospital stay	14.3 (18.6)	10.1 (9.67)	0.036
Perioperative mortality	18 (11.7)	1 (1.7)	0.024
30-Day mortality all treated	72 (46.8)	8 (13.8)	0.001

Presented as numbers (%) and mean (standard deviation)

^a^Missing cases: 30 patients

The postoperative care was prolonged in OR patients (longer ICU, hospital stay), and they had a higher perioperative and 30-day mortality than EVAR treated (Table 2). The 30-day mortality for treated women and men was similar (13/50 vs 48/162, 29 %, *p* = 0.961; Table 1).

#### Untreated

A majority of patients admitted to the ER with rAAA were treated (212/283, 75 %). The mortality among patients who were not operated was 100 %. The untreated patients had a higher mean age than treated [84 (SD 8.20) vs 77 (SD 8.42) years, *p* < 0.001; Table 3]. There was a similar proportion of men and women in the untreated group (*p* = 0.63; Table 1; Fig. 2). A higher proportion of the untreated patients had a previously diagnosed AAA (35/71 vs 50/212, *p* < 0.001). The age difference between women and men is smaller in the untreated group compared to the treated (85.8 vs 83.2 years for untreated and 80.7 vs 75.7 years in treated, *p* < 0.001).

**Table 3:** Preoperative characteristics for treated and untreated, treatment and outcome variables for all included patients

Variable	Untreated *N* = 71(25 %)	Treated *N* = 212(75 %)	*p*
Mean age (SD) years	83.9 (8.20)	76.9 (8.42)	<0.001
Women	19 (27)	52 (25)	0.634
Previously detected	35 (49)	50 (24)	<0.001
Hypertension	42 (59)	133 (63)	0.818
Heart disease	30 (42)	72 (34)	0.444
Diabetes	5 (7)	30 (14)	0.276
COPD	11 (15)	31 (15)	0.967
Blood pressure, lowest registered^a^	67.87 (45.51)	80.58 (41.68)	0.074

Presented as numbers (%) and mean (standard deviation)

^a^Missing cases, in analysis; 19 untreated and 30 treated

## Discussion

This contemporary series on rAAA patients shows that 75 % of rAAA patients admitted to a hospital are treated, and the survival rate among the treated patients is better than reported in older series. There is also a lack of gender differences in crude operation rates or outcome when analyzing all patients admitted with rAAA, although women were considerably older than men. Several recent publications have reported on similar rates of untreated as ours, 25 % and also a lack of gender differences. A better immediate outcome is found in patients treated with EVAR rather than OR.

Few series have historically included the untreated group; however, several recently published series have included them [2, 9, 10, 23]. The report from the UK on rAAA has a similar proportion as Stockholm, 26 % [2]. The recently published Finnish study based on data from 2001 to 2011 report 57 % untreated, however, includes persons dying at home, before treatment is evaluated. Including only the patients admitted to hospital were included, their rate would be similar to ours (25 %, 70/281) [10] (Table 4). The recently published report on patients collected during 8 years, in the Netherlands on “treated versus untreated,” is difficult to interpret and has a parallel ongoing bias for inclusion into the Ajax trial [11]. The Norwegian study covering 2000–2013, on 216 patients, has quite similar data [9] (Table 4). The older, often cited autopsy-based report from Malmö had a higher rate of untreated [29]. Probably, we have a shift in the care of rAAA and AAA patients, and a more positive attitude toward treating more diseased patients. The better immediate results of EVAR versus OR could also contribute [18, 23, 30].

**Table 4:** Previous publications on numbers and outcome for untreated and treated patients admitted with rAAA

Author Time period	Number of patients admitted	Untreated	Mortality all	Mortality treated
Bengtsson et al. (1971–1986)	125	51 %	79 % (30 days)	57 %
McPhee (2001–2004)	37,016	32 % (41 % women vs 30 % men)	Not reported	37 % (43 % women vs 36 % men)^b^
Ozdemir (2005–2010)	9877	42 %	67.5 % (90 days)	44 % (90 days)
Vänni^a^ (2001–2011)	354	26 % (134 nonadmitted)	71 %	43 % treated
Reite (2000–2013)	196 (216)	26 %	64 % (30 days)	51 % (90 days)
Hultgren (2009–2013)	280	25 %	53 % (30 days)	38 % (30 days)

^a^134 died outside hospital (27 %)

^b^In-hospital

Age has always been reported to be an influential factor when outcome is evaluated in surgical series [1, 22, 27, 31, 32]. It is clear that the number of aged patients is high among the untreated in this and other series. In our study, the difference in mean age in untreated and treated was 5 years, which is in the range of 3–6 years found in previous studies [1, 12, 13, 16]. In our study, the age difference between men and women who were untreated was only 2 years, compared to the 5-year age difference in treated, which implies that age is an important factor when the decision to operate or not is made, more than gender.

During the study period, a shift in the care of patients was performed in the County, and an “EVAR first strategy” was developed, which have been reported by others [2–4, 23, 27]. This started in 2010–2011, and specific analysis has therefore not been analyzed in detail. The operation rates for AAA overall have increased for both men and women since the 1970s [1, 3, 13, 16]. During the period from 1971 to 1986, operation rates were reported to be 28 % for women and 56 % for men [29]. Population-based retrospective studies have showed that a lower proportion of women than men with rAAA are operated [13, 16]. The operation rate for women in our study is, however, high and corresponds well to other contemporary reports (Reite 22 %, Dalman 26 %) [9, 23]. This indicates a shift in the care of rAAA patients and will also increase the mean age in treated (Fig. 3). One can suspect that the increasing number of screened men (which started in the fall 2010) will affect the proportion of treated women with rupture, since the number of men with rupture will decrease in the future [8, 33]. The screening program has not yet affected the rupture rate in the general population in our region, so findings from this report should still be applicable to populations without general screening.

The number of elderly patients treated with EVAR is clearly shown in this material, which confirms other reports, both registry based and single centers. The case mix makes all comparisons on outcome difficult between OR and EVAR patients; the EVAR-treated patients are older, but obviously have a different morphology with smaller aneurysms, and require less ICU and hospital stay, as has been shown by others [12, 21, 23].

The Swedvasc annual report 2014 showed that the total proportion of rAAA operations conducted with EVAR in Sweden was 39 % in 2013 [3]. In our study, the total rate of EVAR among operated patients (men and women) during the 5-year period was 27 %, with more performed in the latter period. The largest randomized trial comparing EVAR first strategy versus OR first for rAAA found that women have greater survival benefit from EVAR relatively to OR than men [26].

The outcome in a rAAA must always include reflections upon the number of treated versus untreated, since a high intervention rate will give a poorer survival rate and a lower intervention rate gives a better survival rate due to the selection bias. This is difficult to control, and mean age is a possible surrogate variable for such a biased inclusion. Our study includes all patients with rAAA admitted to hospital, but does not tell us about the number of deaths from rAAA outside of hospital. The in-hospital mortality from rAAA has declined since the 1980s, partly due to the introduction of EVAR [1, 16, 30], but there is no reason to believe that the mortality from rAAA outside of hospital has declined. Bengtsson et al. [29] performed a retrospective study in Malmö, including operated patients who died in hospital and outside of hospital (autopsy rate 85 %). Their poorer outcome compared to reported contemporary data from us and others could reflect a higher awareness today when referring rAAA patients, increased willingness to treat even old patients with comorbidity and possibly the increased use of EVAR (Table 4).

Postoperative mortality has been reported to be higher for women than for men in several previous studies, even if contradictory findings have been reported after age adjustments [5, 12, 13, 16, 21, 31]. In our study, and others, 30-day crude mortality was almost identical between all men and women regardless of the age difference [9, 14, 31]. The reasons mentioned above are probably highly applicable for both women and men.

## Strengths and limitations

The included patients in this study represent a fifth of all admitted persons with rAAA in Sweden. In an international comparison, this is a large material for this type of study, considering the study period. As all retrospective studies, this study has its limitations as we rely on information partly collected in an acute care setting without research purposes, resulting in missing data. The results do harmonize well with other recent reports.

### Causes of death

In Stockholm County, approximately 15,500 persons die annually (15–18 % of the total death rate in Sweden), and the death rate in Sweden is 87,000–90,000 annually. The autopsy rate in Sweden was only 11 % in 2013, and in elderly women 6 %, the reliability of reported Causes of Deaths is therefore questionable [33]. A further extraction of reported deceased persons registered as rAAA at home, from central registries, would probably therefore not bring substantial information.

## Conclusions

Our results and other contemporary series show a shift toward a higher rate of treated persons with rAAA, and improving outcomes for this patient group. The influence of EVAR as a “first-line” treatment modality does contribute to the improved outcome. In contrast to our hypothesis, female sex does not influence the treatment rates or outcome for rAAA patients, rather age is a factor that influences the willingness to treat patients with rAAA. Further analysis of radiological findings and postoperative complications to explain the positive transition in the care of this patient group are called for.
